# Machine learning in 3D auto-filling alveolar cleft of CT images to assess the influence of alveolar bone grafting on the development of maxilla

**DOI:** 10.1186/s12903-023-02706-8

**Published:** 2023-01-11

**Authors:** Xin Zhang, Niu Qin, Zhibo Zhou, Si Chen

**Affiliations:** 1 Department of Orthodontics, Peking University School and Hospital of Stomatology & National Center of Stomatology & National Clinical Research Center for Oral Diseases & National Engineering Research Center of Oral Biomaterials and Digital Medical Devices, Beijing, People’s Republic of China; 2 Department of Oral and Maxillofacial Surgery, Peking University School and Hospital of Stomatology & National Center of Stomatology & National Clinical Research Center for Oral Diseases & National Engineering Research Center of Oral Biomaterials and Digital Medical Devices, Beijing, People’s Republic of China

**Keywords:** Machine learning, CT, Unilateral cleft lip and palate, Alveolar cleft, Alveolar bone grafting, Auto-filling alveolar cleft

## Abstract

**Background:**

Machine learning based auto-segmentation of 3D images has been developed rapidly in recent years. However, the application of this new method in the research of patients with unilateral cleft lip and palate (UCLP) is very limited. In this study, a machine learning algorithm utilizing 3D U-net was used to automatically segment the maxilla, fill the cleft and evaluate the alveolar bone graft in UCLP patients. Cleft related factors and the surgery impact on the development of maxilla were analyzed.

**Methods:**

Preoperative and postoperative computed tomography images of 32 patients (64 images) were obtained. The deep-learning-based protocol was used to segment the maxilla and defect, followed by manual refinement. Paired t-tests and Mann-Whitney tests were performed to reveal the changes of the maxilla after surgery. Two-factor, two-level analysis for repeated measurement was used to examine the different trends of growth on the cleft and non-cleft sides of the maxilla. Pearson and Spearman correlations were used to explore the relationship between the defect and the changes of the maxillary cleft side.

**Results:**

One-year after the alveolar bone grafting surgery, different growth amount was found on the cleft and non-cleft sides of maxilla. The maxillary length (from 34.64 ± 2.48 to 35.67 ± 2.45 mm) and the alveolar length (from 36.58 ± 3.21 to 37.63 ± 2.94 mm) increased significantly only on the cleft side while the maxillary anterior width (from 11.61 ± 1.61 to 12.01 ± 1.41 mm) and posterior width (from 29.63 ± 2.25 to 30.74 ± 2.63 mm) increased significantly only on the non-cleft side after surgery. Morphology of the cleft was found to be related to the pre-surgical maxillary dimension on the cleft side, while its correlation with the change of the maxilla after surgery was low or not statistically significant.

**Conclusion:**

The auto-segmentation of the maxilla and the cleft could be performed very efficiently and accurately with the machine learning method. Asymmetric growth was found on the cleft and non-cleft sides of the maxilla after alveolar bone graft in UCLP patients. The morphology of the cleft mainly contributed to the pre-operation variance of the maxilla but had little impact on the maxilla growth after surgery.

## Introduction

As a congenital maxillofacial hypoplasia, the prevalence of unilateral cleft lip and palate (UCLP) is 6.64 per 10,000 births worldwide and even higher in some developed area, with the deformity of midfacial areas, such as the incomplete formation of lip, alveolar, palate, accompanied by problems of appearance, pronunciation and feeding [1–4].

Alveolar bone grafting (ABG) was of great importance in the cleft lip and palate team approach. It could not only fill the cleft area but also support the alar base, close oroantral fistulae, and help the eruption of canines within the cleft area [5]. ABG included primary alveolar bone grafting (PABG) and secondary alveolar bone grafting (SABG). PABG was rarely used because of its adverse effects on the development of the maxilla [6]. For SABG, the latest cephalometric studies showed that surgeries performed after 2 years of age (and after palatoplasty) had limited negative effect on subsequent maxillofacial development regardless of its timing [7, 8]. However, head films were 2D images, with disadvantages like no volumetric information and structural overlap, and might bring limitation to the research results. More importantly, lateral cephalometric images are not possible to assess the effect of surgery on the cleft side and the non-cleft side respectively. Three-dimensional (3D) computed tomography (CT) has obvious advantages in evaluating asymmetric structures [9]. It had been demonstrated that maxillary asymmetry concentrated in the dentoalveolar area near the cleft and nasal chamber [10–12]. However, few studies explored the effect of SABG on the maxilla development with CT [13].

Besides, it is important to access the volume of cleft accurately by CT before SABG. Firstly, it can avoid taking excessive or inadequate bone and reduce trauma to the donor site [14]. Secondly, the volume of clefts can guide where to take bone. When the requirement of bone is small, the mandibular angle can be the donor site, so there is no need for an extra-oral incision [15]. However, to obtain the accurate cleft volume, most researchers segmented the maxilla and filled the clefts through threshold division and manual modification, time-consumingly and laboriously [5, 16]. Machine learning is a branch of artificial intelligence that uncovers patterns in data automatically and then applies the detected patterns for future data prediction [17]. Machine learning based auto-segmentation of 3D images has been developed rapidly in recent years. However, the application of this new method in the research of patients with unilateral cleft lip and palate (UCLP) is very limited.

In this study we used deep-learning-based protocol, to segment the maxilla and the cleft automatically in CT of SABG patients. This is continuation of method of our previous article about “Machine learning in 3D auto-filling alveolar cleft of CT images” [18–20]. The aims of this study were to answer the following questions (1) to evaluate the impact of SABG on maxillary development three-dimensionally; (2) to analyze the association between the size of the cleft and the preservation of the grafted bone; (3) to analyze the association between the preserved grafted bone and 3D development of maxilla after the operation.

## Methods

### Subjects

This retrospective study was approved by the Institutional Ethical Committee of Peking University School and Hospital of Stomatology (PKUSSIRB-202280135) and conducted in accordance with the tenets of the Declaration of Helsinki. Informed consent was obtained from all patients before the CT was taken.

Firstly, we performed the retrospective screening in the archived database for all patients with UCLP who had pre-surgery (T1) and one-year post-surgery (T2) CT from 2012 to 2020. The CTs were taken for surgery planning and treatment effect evaluation. The inclusion criteria were as follows:

1. All of the subjects had been diagnosed with non-syndromic UCLP and received primary lip and palate repair.
2. The subjects had never undergone previous alveolar bone grafts, orthodontic treatment, maxillofacial neoplasia, trauma, or orthognathic surgery.
3. The subjects were 8–11 years old.
4. CT before and 1 year after SABG were accessible.

In total, we included 32 patients (64 Spiral CTs), consisting of 17 males and 15 females, with a mean age of 9.59 ± 0.97 years (age range: 8–11 years). The CT images were taken at two different times (T1-T2). The mean difference between T1 and T2 was (11.86 ± 0.81) months.

The CT machine (Optima CT520 series, GE MEDICAL SYSTEMS, American) was used under a standard scanning protocol: 120 kV, 14 mAs, 64 × 16.5 cm field of view, and 1.25 mm slice thickness.

### Maxilla and defect segmentation

The 64 CT images were saved as Digital Imaging and Communication in Medicine (DICOM) files. Our team had adopted 3D U-net as the neural network architecture and used the cross-entropy loss as the loss function to train a maxi segmentation model of normal people and UCLP patients. The Dice similarity coefficient (DSC) of our segmentation model of maxi and cleft segment had been reported to reach 88% and 83% [18–20].

### Description of measurement

**Fig. 1:**
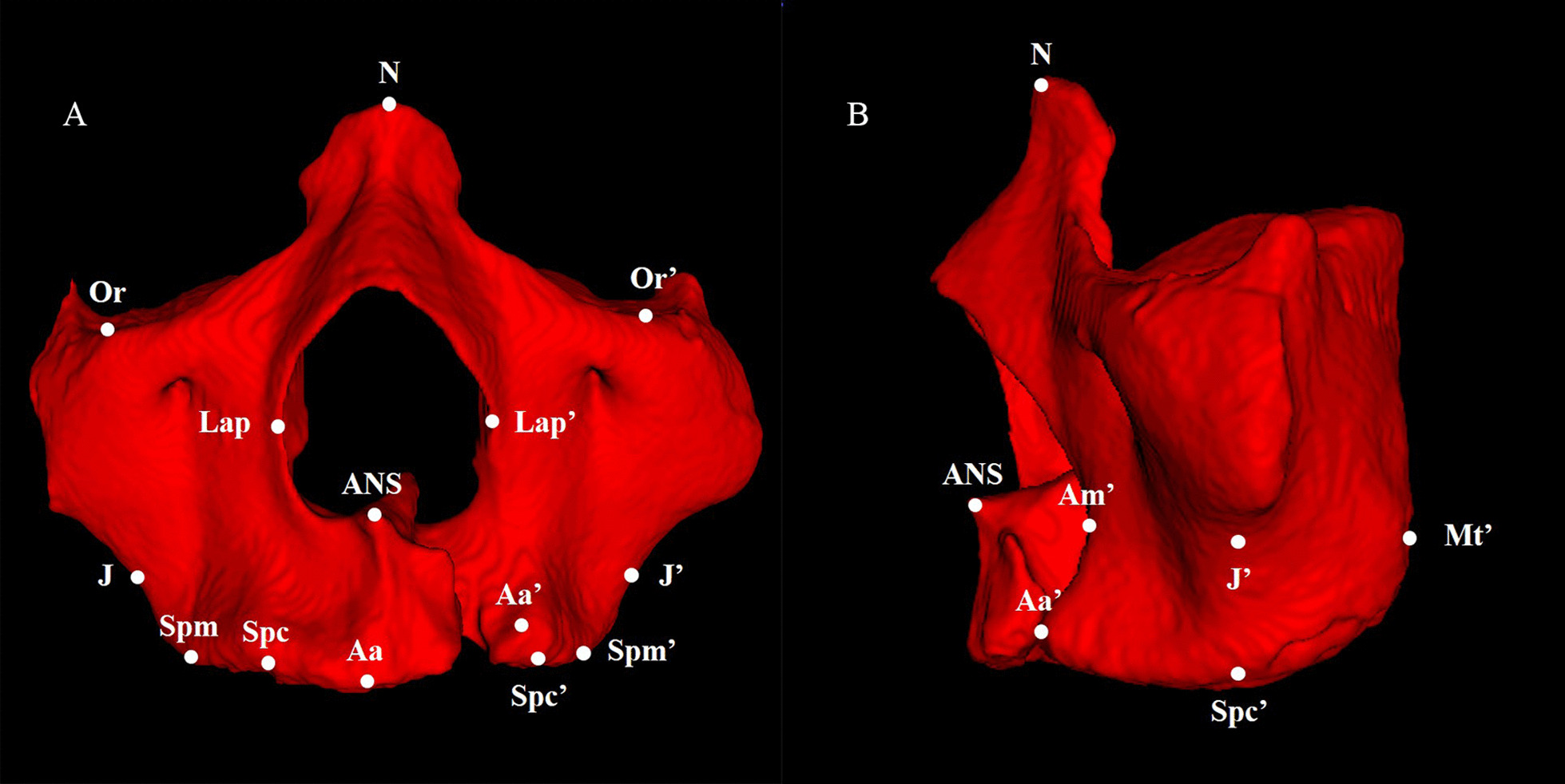
The main landmarks on the 3D segmented model. **A** Frontal view (cleft side and non-cleft side; landmarks on the non-cleft side are denoted by superscript); **B** Lateral view (cleft side);

For 3D morphometric quantification, we determined landmarks by referring to previous literatures [10] and made some adjustments according to the characteristics of Spiral CT, as defined in Table 1 and illustrated in Figure 1A, B. Landmarks were identified on the surface of the 3D segmented model and verified in the multiple planar reformat mode. Three reference planes (the Frankfort horizontal plane, midsagittal plane, and coronal plane) were established as a coordinate system. The maxilla was separated by the midsagittal plane to calculate the volume of the cleft and non-cleft sides respectively. To avoid the impact of teeth on volume, we erased the teeth before calculating maxilla volume. All parameters are defined in Table 1.

**Table 1:** 3D cephalometric landmarks, reference planes and measurements of the maxilla and defect

Items	Definition
*Landmarks*	
N	Intersection of internasal suture with nasofrontal suture
S	Midpoint of sella turcica
ANS	Most anterior point of anterior nasal spine
Po	Uppermost point on bony external auditory meatus
Or	Lowest point on infraorbital edge
Lap	Most lateral points on the nasal aperture
J	Intersection of the outline of the tuberosity of the maxilla and zygomatic buttress
Mt	Posterior most extent of the maxillary tuberosity
Am	Posterior most extent of the anterior contour of the maxilla
Spc	Midpoint of labial alveolar crest of maxillary canine (No missing canine was observed in all subjects)
Spm	Midpoint of buccal alveolar crest of maxillary first molar
Aa	Most inferior anterior point of alveolar crest
*Reference planes*	
Horizontal Plane (FH Plane)	Plane that passes through the bilateral Po and Or on the non-defect side
Midsagittal plane (MS Plane)	Plane perpendicular to the FH plane passing through the N and S
Coronal plane (CR plane)	Plane perpendicular to the FH and MS plane passing through the N

The length, width and height were measured by calculating the distances between the position (voxel coordinates) of landmarks, and the volumes of the maxilla and defect were measured based on the segmentation voxel counting. These measurements were carried out using ITK-SNAP [21] (version 3.8.0; www.itksnap.org).

### Data collection and analyses

All measurements were conducted by two trained examiners. The intraclass correlation (ICC), is greater than 0.96, confirming the consistency of the measurements.

The data were presented as mean values and standard deviations.

When the data showed a normal distribution, t-tests were used to compare different sides at the same time, or the same side before and after operation; otherwise, Mann-Whitney tests were used. Two-factor, two-level analysis for repeated measurement (r-ANOVA) was used to examine whether the change of the cleft side was the same as non-cleft sides after the operation. Pearson and Spearman correlations were used to explore the relationship between the defect and the maxillary cleft side. All statistical analyses were performed using SPSS (Version 25.0; IBM Co.). The level of significance was set at *P* < 0.05.

## Results

### Measurement and analysis of UCLP maxilla and defect

The parameters of the defect structure were shown in Table 2. The analysis and measurement of the cleft and non-cleft sides of maxilla before and one-year after SABG (T1 and T2) were shown in Table 3. Significant differences of maxilla dimension were noticed between the cleft and the non-cleft sides before the surgery, including length and width of maxilla and alveolar bone, anterior alveolar height and the whole maxilla volume. After the surgery, among the above parameters, only the posterior width and alveolar anterior width of maxilla became insignificantly different between the cleft and non-cleft sides. The anterior maxillary height, posterior maxillary height, maxillary volume, and posterior alveolar height increased significantly on both the cleft and non-cleft sides. The maxillary length and alveolar length increased significantly only on the cleft side. The anterior maxillary width and posterior maxillary width increased significantly only on the non-cleft side.

**Table 2:** Defect structure parameters and measurements

Parameter	Mean	SD	Time
L_def_ (mm)	20.08	4.51	T1
W_def_ (mm)	16.98	3.16	T1
H_def_ (mm)	15.53	2.69	T1
V_def_ (mm^3^)	1013.26	332.31	T1
V_bone_ (mm^3^)	569.90	319.98	T2
P_bone_	57.43%	32.28%	–

Defect length (L_def_): Maximum Sagittal distance of the defect;

Defect width (W_def)_: Maximum transverse distance of the defect;

Defect height (H_def_): Maximum vertical distance of the defect;

Defect volume (V_def_): Volume of the segmented defect;

Bone increased volume (V_bone_): Volume of the increased bone in defect area;

Percentage of bone filled (P_bone_): V_bone_/ V_def_ × 100;

**Table 3:** Data measurement and analysis of the cleft and/or non-cleft sides of maxilla before operation (T1) and/or after operation (T2)

Parameter	Time	Non-defect side	Defect side	Compare non-defect side with defect side (P-value)		Compare T1 with T2 (P-value)		r-ANOVA
				T1	T2	Non-defect	Defect	(*P*-value of time*side)
L_max_ (mm)	T1	40.59 ± 2.88	34.64 ± 2.48	0.00*	0.00*	0.56	0.00*	0.00*
	T2	40.49 ± 2.77	35.67 ± 2.45					
AntW_max_ (mm)	T1	11.61 ± 1.61	13.12 ± 1.27	0.00*	0.00*	0.00*	0.23	0.00*
	T2	12.01 ± 1.41	12.86 ± 1.38					
PosW_max_ (mm)	T1	29.63 ± 2.25	30.98 ± 2.03	0.00*	0.32	0.00*	0.42	0.00*
	T2	30.74 ± 2.63	31.15 ± 2.39					
AntH_max_ (mm)	T1	20.61 ± 2.56	20.24 ± 2.86	0.06	0.05	0.00*	0.00*	0.63
	T2	21.63 ± 2.72	21.13 ± 3.11					
PosH_max_ (mm)	T1	26.37 ± 2.61	26.35 ± 3.04	0.94	0.32	0.00*	0.00*	0.87
	T2	27.61 ± 2.76	27.25 ± 2.53					
L_alv_ (mm)	T1	41.52 ± 3.61	36.58 ± 3.21	0.00*	0.00 *	0.06	0.00*	0.04*
	T2	41.88 ± 3.66	37.63 ± 2.94					
AntW_alv_ (mm)	T1	17.6 ± 2.26	19.02 ± 2.19	0.02*	0.33	0.33	0.14	0.08
	T2	17.81 ± 2.22	18.45 ± 2.51					
PosW_alv_ (mm)	T1	27.42 ± 2	29.09 ± 1.72	0.00*	0.00 *	0.88	0.50	0.54
	T2	27.44 ± 2.02	28.98 ± 1.78					
AntH_alv_ (mm)	T1	16.04 ± 3.04	15.38 ± 3.41	0.03 *	0.00 *	0.30	0.89	0.43
	T2	16.46 ± 3.15	15.42 ± 3.42					
PosH_alv_ (mm)	T1	11.66 ± 2.64	11.64 ± 2.76	0.96	0.63	0.00 *	0.00 *	0.73
	T2	12.73 ± 2.73	12.6 ± 3.06					
V_max_ (cm^3^)	T1	18.26 ± 2.47	16.73 ± 2.47	0.00 *	0.00 *	0.00*	0.00*	–
	T2	18.90 ± 2.57	17.49 ± 2.50					

All data of parameters were shown in the form of “Mean ± Standard deviation”

Paired samples t-test analysis or Mann–Whitney tests was used to analyse the cleft and/or non-cleft sides of maxilla before operation (T1) and/or after operation (T2); Two-factor, two-level analysis for repeated measurement (r-ANOVA) was used to analyse the interaction between the sides of maxilla and time points;

^*^Significant at *P*-value < 0.05;

**Fig. 2:**
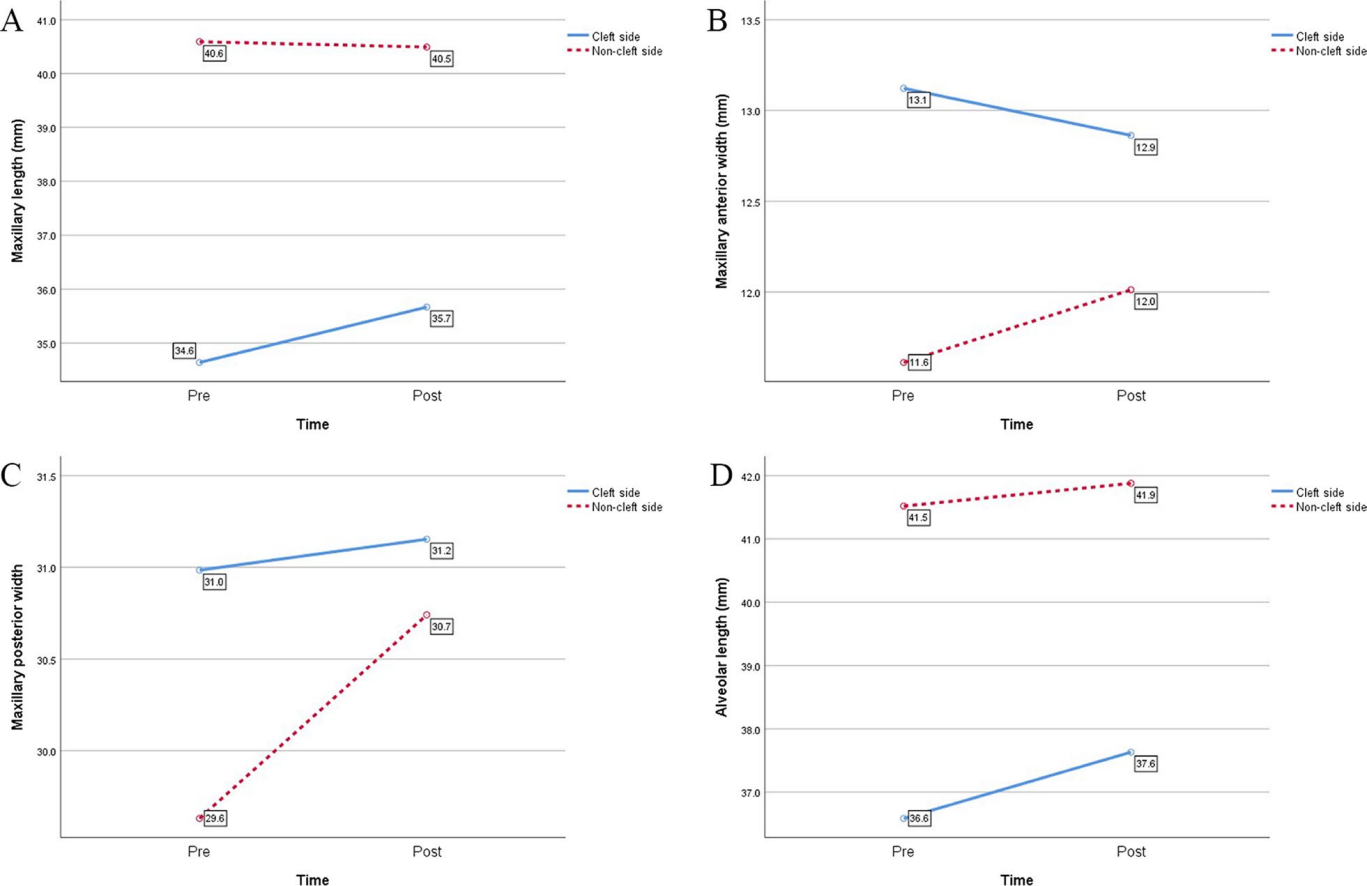
**A** The trend of maxillary length overtime on the cleft and non-cleft sides. The length of the cleft sides increased significantly while that on non-cleft sides changed insignificantly. **B** The trend of maxillary anterior width overtime on the cleft and non-cleft sides. The anterior width of the non-cleft sides increased significantly while that on cleft sides changed insignificantly. **C** The trend of maxillary posterior width overtime on the cleft and non-cleft sides. The posterior width of the non-cleft sides increased significantly while that on cleft sides changed insignificantly. **D** The trend of alveolar length overtime on the cleft and non-cleft sides. The length of the cleft sides increased significantly while that on cleft non-sides changed insignificantly

Maxillary length (L_max_): Sagittal distance from Am to Mt;

Maxillary anterior width (AntW_max_): Transverse distance from Lap to the MS plane;

Maxillary posterior width (PosW_max_): Transverse distance from Lap to the MS plane;

Maxillary anterior height (AntH_max_): Vertical distance from Or to ANS;

Maxillary posterior (PosH_max_): Vertical distance from Or to J;

Maxillary volume (V_max_): Volume of the segmented individual maxilla;

Alveolar length (L_alv_): Maximum sagittal distance from Aa to Mt;

Alveolar anterior width (AntW_alv_): Transverse distance from Spc to the MS plane;

Alveolar posterior width (PosW_alv_): Transverse distance from Spm to the MS plane;

Alveolar anterior height (AntH_alv_): Vertical distance from Spc to ANS;

Alveolar posterior (PosH_alv_): Vertical distance from Spm to PNS;

The results of two-factor, two-level analysis for repeated measurement (r-ANVOA) of the maxilla were also shown in Table 3. Since new bone had been grafted in the maxillary cleft, the maxillary volume was not included in the two-factor, two-level analysis. The P values of maxillary length, anterior width, posterior width, and alveolar length were less than 0.05, indicating that there was an interaction between different sides and time effects. There was different growth trend on the cleft and non-cleft sides in these items after surgery (Fig. 2).

### Correlation between maxillary and defect

When the data showed a normal distribution, Pearson correlation was used to explore the relation between parameters; otherwise, Spearman was used. The results were shown in Table 4.

**Table 4:** Pearson and Spearman correlation analysis of defect and maxilla on the cleft side

	V_def_	L_def_	W_def_	H_def_	V_bone_	P_graft_
V_def_	–	0.635**	0.460**	0.422*	–	–
V_bone_	0.356*	0.408*	0.098	0.252	–	–
P_graft_	–	− 0.06	− 0.132	− 0.034	–	–
V_max_	0.027	0.061	0.076	0.482**	–	–
L_max_	− 0.063	− 0.119	0.111	0.157	–	–
AntW_max_	0.087	0.012	0.399*	0.145	–	–
PosW_max_	0.158	0.037	− 0.029	0.348	–	–
AntH_max_	0.438*	0.462**	0.311	0.402*	–	–
PosH_max_	0.620**	0.404*	0.461**	0.635**	–	–
L_alv_	0.065	− 0.070	0.180	0.316	–	–
AntW_alv_	0.249	− 0.051	0.386*	0.331	–	–
PosW_alv_	0.097	0.081	− 0.158	− 0.063	–	–
AntH_alv_	0.221	− 0.155	0.014	0.380*	–	–
PosH_alv_	0.285	0.103	0.201	0.484**	–	–
ΔL_max_	0.257	0.223	0.277	0.092	0.179	0.06
ΔAntW_max_	− 0.211	0.118	0.071	− 0.076	− 0.022	− 0.174
ΔPosW_max_	− 0.169	− 0.145	0.335	− 0.072	− 0.093	− 0.031
ΔAntH_max_	0.163	0.021	0.371*	0.011	0.028	0.13
ΔPosH_max_	− 0.192	− 0.127	− 0.038	− 0.258	− 0.233	− 0.101
ΔL_alv_	− 0.257	− 0.110	− 0.170	− 0.058	0.035	0.191
ΔAntW_alv_	0.072	− 0.118	0.025	0.002	− 0.107	− 0.165
ΔPosW_alv_	0.143	− 0.109	0.272	0.037	− 0.138	− 0.196
ΔAntH_alv_	0.059	0.108	0.121	0.131	− 0.050	− 0.212
ΔPosH_alv_	− 0.293	− 0.294	− 0.279	− 0.014	0.143	0.284

Δmeans the change of the parameter after the operation

^*^Significant at *P*-value < 0.05; **Significant at *P*-value < 0.01

## Discussion

This study applied machine learning method to segment the maxilla and the cleft automatically in CT of UCLP patients before and 1-year after SABG surgery to quantify the defect related maxillary morphological characteristics and post-surgery growth change. We optimized the 3D U-net with a non-rigid registration technique and combined it with manual refinement, making it satisfied with the clinical study [18–20]. Based on this technique, auto-segmentation of one sample only took several minutes.

**Fig. 3:**
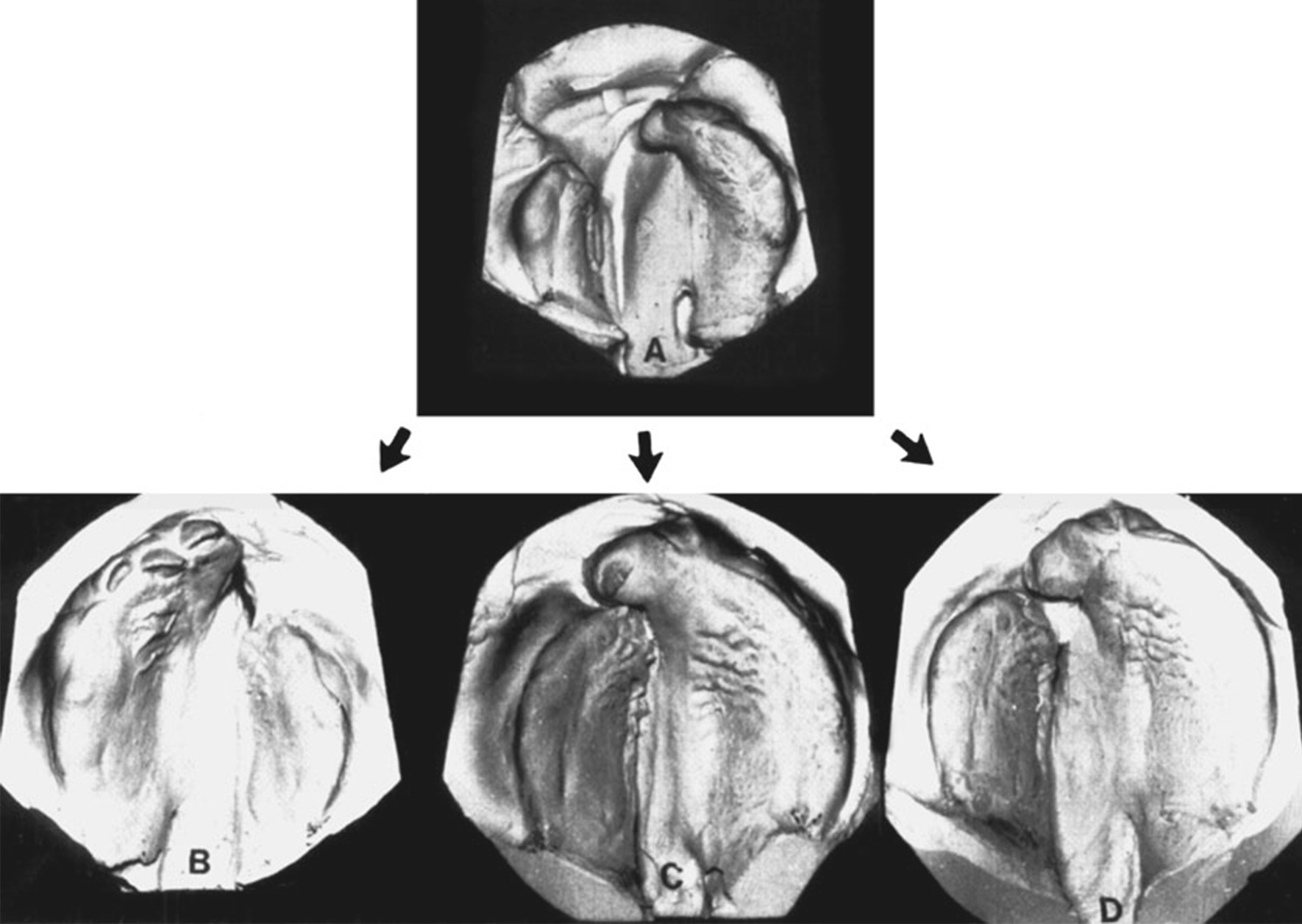
Complete unilateral cleft lip and palate (CUCLP) before (**A**) and after (**B**) lip surgery. With the establishment of muscle continuity, the lesser segment moves medially, while the premaxillary portion of the larger segment moves medio-inferiorly, both acting to reduce the cleft width. Any of the following segmental relationships can result. **B** No contact between segments. The inferior turbinate on the cleft side makes premature contact with the bowed nasal septum. **C** The premaxillary portion of the larger segment overlaps the smaller segment. **D** The segments form a butt joint showing good approximation. (Both figures and notifications come from ref [23])

Based on the segmentation results, morphological dimensions of maxilla and cleft were measured. Different growth was noticed between the cleft and non-cleft sides. The maxillary length, maxillary volume, alveolar length, and alveolar anterior height on non-cleft sides were larger than those of cleft sides. The anterior and posterior width of the maxilla on cleft sides were larger than those on non-cleft sides. The above results of our study agreed with the previous studies [10, 12]. For those asymmetries, Agarwal et al. [22] explained it based on the dysplasia of UCLP: the bone clefts made the lateral incisors and/or the canines absent in the defect sides, along with the alveolar missing and the premaxillary portion of the defect side was skewed along with the incisors, which shortened the alveolar vertical height and maxillary and alveolar length; the deformed pyriform margin derived from the attachment of the accessory cartilages and the lateral crus served to pull down the nose further caudally, leading to a tilting of the nasal tripod toward the cleft sides and then the width of maxillary increased. However, the anterior and posterior alveolar widths on cleft sides were larger than those of non-cleft sides, which was different from previous studies. Li [12] reported that the means of anterior and posterior alveolar widths on defect sides were larger than those of non-defect sides, but the difference was not significant. Wang [10] reported those indicators of cleft sides were smaller. The reason why our result on alveolar width was different from previous studies might due to individual differences in the self-healing of alveolars after repairing cleft lips. Berkowitz stated in his treatise [23] that intraoral and extraoral muscles worked together to ensure the normal development of the maxilla during the growth of the embryo. Extraoral muscles lost their continuity in UCLP, resulting in the lateral pull of the cleft sides by the abnormal labio-buccal muscles, and at the same time, the tongue facilitated the abnormal lateral movement. After repairing the cleft lip, the continuity of the perioral muscles was restored, and the cleft sides moved inward to reduce the width of the maxilla. But the alveolar inward movement might vary from person to person [24] (Fig. 3), resulting in disagreements among researchers.

Many researchers used lateral cephalograms to explore the development of the maxillary of UCLP, and the effect of SABG on the maxilla. Berkowitz concluded [23] that the length of the maxilla increased rarely from 5 to 18 years old and negated the SABG had a deleterious effect on the growth of midface. For the limitation of lateral cephalograms, we cannot measure the cleft and non-cleft sides separately. Therefore, in this study, CT was used to explore the impact of SABG on maxillary growth and development for both sides respectively.

According to the results of this study, the change of the maxillary length, the anterior and posterior width of maxillary, and the alveolar length on cleft sides were different from those on non-cleft sides one year after surgery. The maxillary and alveolar lengths on the non-cleft sides were significantly longer than those on cleft sides at both pre-operative and post-operative time points. However, compared to pre-operation, these lengths of the non-cleft sides changed insignificantly after surgery which was consistent with the above Berkowitz’s conclusion. But these lengths on cleft sides increased significantly, indicating that the cleft side tend to have a catch-up growth in length after the surgery. SABG had a positive effect on the length growth of both the maxilla and alveolar on the cleft sides.

Anterior and posterior widths of the maxilla on non-cleft sides increased significantly within 1 year after surgery, while these widths of the cleft sides had no significant change after treatment. These differences indicated the overall transversal maxillary growth of the cleft sides were lower than those of the non-cleft sides. The maxillary anterior and posterior widths were larger on the cleft sides. This may related to lateral transposition of the cleft segment. However, the difference of the maxillary posterior width on the cleft and non-cleft sides became insignificant one-year after surgery. This may come from the reason that the degree of deformity of the maxillary posterior width on cleft sides was less than that of maxillary anterior width so that when the posterior maxilla of non-cleft side became wider, the width on both sides tended to be the same. The alveolar anterior and posterior widths on two sides changed insignificantly after surgery. However, an increase trend of mean was found on the non-cleft side and a derease trend of mean on the cleft side. Since the anterior alveolar width became larger along with the eruption of the canine, the insignificant change indicated that SABG might have limited benefit to the transverse growth of the alveolar bone, especially to the anterior alveolar bone.

As for the height of maxilla and alveolar bone, about 1 mm increase was noticed one-year after the surgery, except for the anterior alveolar height. On the cleft side, this height was significantly lower than that of the non-cleft side at both T1 and T2. This result indicated that SABG had limited effect on the vertical growth of the alveolar bone on the cleft side. One year after the surgery, the volume of maxilla on both sides increased due to growth and grafting, whereas the volume of the cleft side was still smaller than that of the non-cleft side, indicating that the SABG could not eliminate the defect completely.

Brudnicki showed in the latest papers that SABG indeed increased the volume of alveolars but had an adverse effect on the maxilla growth, although might be limited and regardless of timing [7, 8, 13]. Combined with the results of this study, we further speculated that on one hand, SABG increased the volume and length of the maxilla, especially on the cleft sides; on the other hand, the transversal growth of the maxilla on the cleft sides was more negatively affected by SABG than the non-cleft sides. Since the traversal deficiency could be resolved by orthodontic maxilla expansion, maybe it is better to perform SABG at earlier age than generally assumed to be optimal for subsequent maxillary growth.

Although it is of challenge to determine factors that contribute to maxillary variability, the cleft on the maxilla has been considered as one factor of maxillary dysplasia. In our study, the length of the cleft was found to have the strongest relationship with the volume of the cleft. However, the correlation between increased bone in the cleft region or percentage of bone fill and the morphology of the cleft (length, width, height, and volume) was not high or even had no statistical significance, which was in accordance with previous papers [9, 25–27]. The growth of height of the cleft contributed to the increase of volume of maxilla, anterior height, and posterior height of alveolar ridge. And the anterior width of both maxilla and alveolar ridge was positively associated with the width of the cleft. All of these agreed with the previous study [10]. Besides, we also found that the anterior and posterior heights of maxilla were associated with the morphology of the cleft at a medium level. In terms of the change of maxillary and alveolar parameters after the SABG surgery, almost no change was related to the morphology of the cleft, the bone increased, and the percentage of bone fill.

The limitations of the present study included its retrospective character and a relatively small number of participants. More importantly, there was no control group. Although we compared the cleft sides to the non-cleft sides to explore the changes of cleft sides after surgery, the growth of non-cleft sides could not represent that of UCLPs at the same age without receiving SABG. And the growth of UCLP may differ from the normal children at the same age, so it would be better to have a control group of normal children at the same age, which was hard to achieve due to ethical reasons. In addition, the one-year follow up for the assessment was short and CBCT could be better than CT for bone evaluation.

## Conclusions

Cleft and non-cleft sides of the maxilla have different growth trends after alveolar bone graft in UCLP patients. The growth amount of the maxillary width on the cleft side is smaller than that on the non-cleft side. Bone graft surgery is beneficial to the length growth of the maxilla and alveolar ridge but has limited benefit to the width and height of the maxilla and the alveolar width on the cleft side. The morphology of the cleft contributes to the variability of the maxilla, while has almost no effect on the percentage of the preserved grafted bone and the growth change of the maxilla after the surgery.

## Data Availability

The datasets generated and analysed during the current study are not publicly available due to compromised individual privacy but are available from the corresponding author on reasonable request.
